# Plate Fixation for Irreducible Proximal Humeral Fractures in Children and Adolescents—A Single-Center Case Series of Six Patients

**DOI:** 10.3390/children8080635

**Published:** 2021-07-26

**Authors:** Florian Freislederer, Susanne Bensler, Thomas Specht, Olaf Magerkurth, Karim Eid

**Affiliations:** 1 Department of Orthopaedics and Traumatology, Kantonsspital Baden (KSB), Im Ergel 1, 5404 Baden, Switzerland; thomas.specht@ksb.ch; 2 Unit for Musculoskeletal Radiology, Department of Radiology, Kantonsspital Baden (KSB), Im Ergel 1, 5404 Baden, Switzerland; susanne.bensler@ksb.ch; 3 Unit for Pediatric Radiology, Department of Radiology, Kantonsspital Baden (KSB), Im Ergel 1, 5404 Baden, Switzerland; olaf.magerkurth@ksb.ch

**Keywords:** humerus fracture, proximal humeral fracture, children, plate fixation, ORIF, tissue entrapment, biceps

## Abstract

Background: Recommended treatment for severely displaced proximal humeral fractures in children is the closed reduction and percutaneous fixation by K-wires or intramedullary nailing. Methods: From January 2016 to January 2017 6, 21 children/adolescents (range 8 to 16 years) with proximal humeral fractures were treated surgically for severe displacement. In these six patients, several attempts of closed reduction were unsuccessful, and an open reduction was performed. The humeral head was fixed with a 3.5 mm T-plate without affecting the growth plate. Plate removal was performed at a mean interval of 132 days after initial surgery. Two years after initial surgery, the clinical outcome was assessed by the Constant–Murley score and QuickDASH score (including sport/music and work) and the shoulder joint was evaluated with a standardized sonographic examination for the rotator cuff and the conjoint tendon. Results: In all six patients, dorsal displacement of the fracture was irreducible due to the interposition of tendinous or osseous structures. Intraoperatively, the interposed structures were the long biceps tendon in two, periosteal tissue in two, a bony fragment in one, and the long biceps tendon together with the conjoint tendon in one case. At mean follow-up of 26 months (range 22 months to 29 months), patients showed very good clinical results with an excellent mean Constant–Murley score of 97.5 (range 91 to 100) and mean QuickDASH score (including sport/music and work) of 5.5 (range 0–20.8). An X-ray follow-up 6 weeks after surgery demonstrated early consolidation and correct alignment in all patients. A sonographic evaluation at 2 years post injury showed that the biceps and the conjoined tendon were intact in all patients. Conclusions: If a proximal humeral fracture is not reducible by closed means, a tissue entrapment (most likely biceps tendon) should be considered. Treatment with an open reduction and plate fixation yields very good clinical and radiological results and preserves interposed structures as the biceps and conjoint tendon.

## 1. Introduction

Proximal humeral fractures in children and adolescents are rare injuries, representing less than 5% of all pediatric fractures with a peek incidence between the age of 11 and 15 years [1,2,3]. These fractures can be physeal or metaphyseal. Metaphyseal fractures account for about 70% of the cases [4]. The muscle attachments of the rotator cuff proximally, and of the deltoid, as well as of the pectoralis major distally, are responsible for the specific fracture pattern.

**Figure 1:**
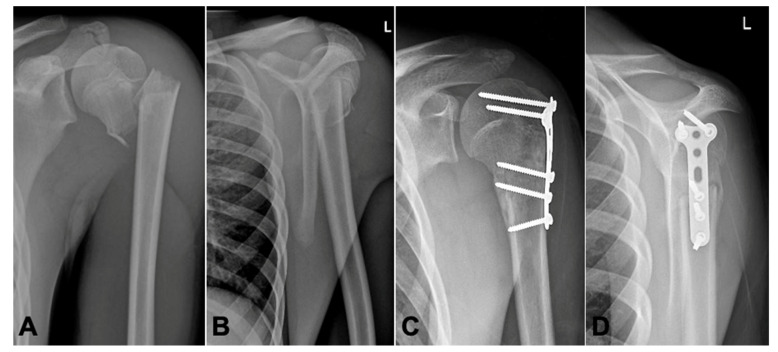
Radiographic shoulder views (Pat. N° 4) (ap/Neer). (**A**,**B**): Dorsally displaced humeral head. (**C**,**D**): 10 weeks after open reduction and plate fixation.

Muscular tension displaces the proximal fragment in varus and posteromedially, whereas the distal fragment moves anteriorly and in adduction (Figure 1A,B).

Another explanation for the anterior displacement of the distal fragment might be the thinner and weaker anterior periosteum [5].

Neer and Horwitz classified proximal humeral fractures in children in four grades according to the severity of displacement (I: <5 mm; II: <1/3 shaft width; III: <2/3 shaft width; IV: >2/3 shaft width) [2].

Most fractures of the proximal humerus in the skeletally immature are not or only minimally displaced and can be treated conservatively [4,6,7].

The management of severely displaced proximal humeral fractures in children is still controversial [3]. Most of the displaced proximal humeral fractures (Neer and Horwitz ≥ III) are treated by closed reduction and intramedullary nailing or by percutaneous fixation with K-wires [2,8,9]. Due to the high remodeling potential, moderate malalignment after closed reduction is acceptable. In addition, since around 80% of the longitudinal growth of the humerus results from the proximal physis, any mechanical interference with the growth plate by implant materials is usually avoided [10,11].

However, insufficient alignment after closed reduction may require open reduction and stable fixation which has already been described 40 years ago by Weber with an excellent clinical outcome [4,12,13,14].

In our institution, closed reduction and pin fixation are routinely performed to address displaced pediatric proximal humerus fractures. If secondary displacement is observed or primary reduction is not satisfactory, presumably due to tissue entrapment, open reduction and internal fixation are performed.

The aim of the study is to assess clinical and radiological results after an open reduction, plate fixation and plate removal of irreducible displaced proximal humeral fractures in older children and to evaluate the functional integrity of the tissue—mainly the long head of the biceps tendon—interposed in the irreducible fracture.

## 2. Material and Methods

Between January 2016 and January 2017, 21 skeletally immature patients with proximal humeral fractures were treated at the authors institute (level 1 trauma hospital). Six of these patients (29%) were treated by open reduction and internal fixation. All of these six patients were included in the study.

The inclusion criteria were: available standard x-rays in anteroposterior (AP) and Neer view preoperatively and 6 weeks postoperatively, severely displaced humeral fracture (Neer–Horwitz III/IV), not reducible by closed means, open physis and hardware removal performed. After 2 years, patients were prospectively assessed with a clinical and sonographic examination. Informed consent was obtained from the patient’s or her/his legal representative.

Approval by the Ethical committee Nordwestschweiz Nr. 2018-01405 was obtained.

### 2.1. Surgical Technique

All of the operative procedures were carried out under general anesthesia. The patients were positioned in beach chair position. An initial attempt of closed reduction was conducted in all patients. This was performed by gentle longitudinal traction with abduction and external rotation of the arm. An image intensifier was used to monitor reduction. If closed reduction failed, the surgeon proceeded with open reduction by means of an anterior deltopectoral approach. Entrapped tissue or periosteum were gently freed. Once reduction was achieved, a 3.5 mm T-plate was contoured on the anterolateral part of the proximal humerus and temporary K-wires were inserted through the plate holes to hold the reduction. Under image intensifier, attention was drawn not to injure the physeal plate by the drill or the screws. After confirming anatomical reduction, the K-wires were then replaced with conventional cortical screws.

Surgical wounds were closed with absorbable VICRYL (Ethicon, Raritan, NJ, USA; Johnson & Johnson, New Brunswick, NJ, USA) sutures. Skin was closed using absorbable MONOCRYL (Ethicon, Raritan, NJ, USA; Johnson & Johnson, New Brunswick, NJ, USA) sutures.

Postoperatively, a brace (type Gilchrist) was applied for 2 weeks. For the first 6 weeks, range of motion was limited to 90° abduction and flexion. For the first 2 weeks, only passive mobilization out of the brace was allowed, followed by actively assisted movements.

### 2.2. Clinical Assessment

Patient outcome was assessed by clinical and sonographic evaluation. The clinical examination was carried out by a single independent observer and sonography by a single radiologist specifically trained in musculoskeletal imaging. The Constant–Murley score and QuickDASH (Disability of the Arm, Shoulder and Hand) score (including sport and music/work modules) were used for objective assessment [15,16]. The Constant–Murley score is a 100-point shoulder score, which assesses the range of motion of the treated shoulder joint. Forward flexion, extension, abduction, and low (arm in adduction) and high (arm in 90° of abduction) external and internal rotation were measured using the standardized neutral-zero method in degrees. A goniometer was used for the measurement. The abduction strength was measured using a spring balance (Macro-Line 80020, Fa. Pesola), which was attached distally on the forearm adjacent to the wrist with the method described and validated by Bankes et al. [17]. Strength was measured with the arm in 90 degrees abduction, full extension of the elbow, and the palm of the hand in pronation. The patient was asked to maintain this position for five seconds. This procedure was repeated three times, with at least a one-minute time interval. The average in kilograms (kg) was noted. The same procedure was performed with the contralateral arm. The measurement should be pain free. If pain was present or the patient was unable to abduct above 90°, the score equaled zero. The strength score was calculated from the highest score of the three attempts. The score corresponds to the force in kilogram.

We also performed this procedure with the contralateral arm to obtain the individual Constant score as described by Fialka et al. [15]. For interpretation, the results of the Constant–Murley score were divided into four subscales: excellent 90–100; satisfactory 80–89; unsatisfactory 70–79; failure > 70. The QuickDASH outcome measure was a 100-point shoulder score. It measured a 30-item (+4 sports/music, +4 work) questionnaire of physical and social function with symptoms in any or all joints in the upper extremity. The lower the DASH score, the better the outcome.

In addition, medical records were reviewed. All patients had a normal shoulder function and no previous operations on the affected side. Radiological evaluation included standard anteroposterior and Neer view of the shoulder. Follow-up radiographs were carried out six weeks and between two and three months postoperatively. Subsequently, hardware removal was performed.

### 2.3. Ultrasound Imaging

All patients were scanned in a sitting position with a relaxed arm hanging freely on the side. For the examination, a GE LOGIQ E9 ultrasound system (GE Healthcare; Chicago, IL, USA) with a linear transducer with a bandwidth of 6–15 MHz was used.

The tendons of the subscapularis, supraspinatus and infraspinatus tendon were examined along their long and short axis.

The subscapularis tendon was examined with the arm externally rotated and the elbow fixed at the iliac crest. For the evaluation of the supraspinatus tendon, the patient’s arm was placed posteriorly, with the palmar side of the hand on the superior aspect of the iliac wing with the elbow flexed and directed posteriorly. To examine the infraspinatus tendon, the arm was placed anteriorly with the hand on the opposite shoulder.

The long head of the biceps tendon was examined along the long and short axis with the arm placed in slight internal rotation. The integrity of the conjoint tendon was examined also in both planes with the arm placed in external rotation.

## 3. Results

There were five boys and one girl with a mean age of 14 years (8–16 years). At the time of injury, mostly accidents during physical activities which caused an isolated injury to the proximal humerus, all fractures were proximal metaphyseal fractures. Five patients had a Neer–Horwitz Grade III fracture and one had a Grade IV (patient N° 4, see Table 1) completely displaced fracture of the proximal humerus (Figure 1A,B). In these six patients, a closed reduction was attempted; in five patients, an immediate conversion to open surgery with open reduction and internal plate fixation was necessary.

**Figure 2:**
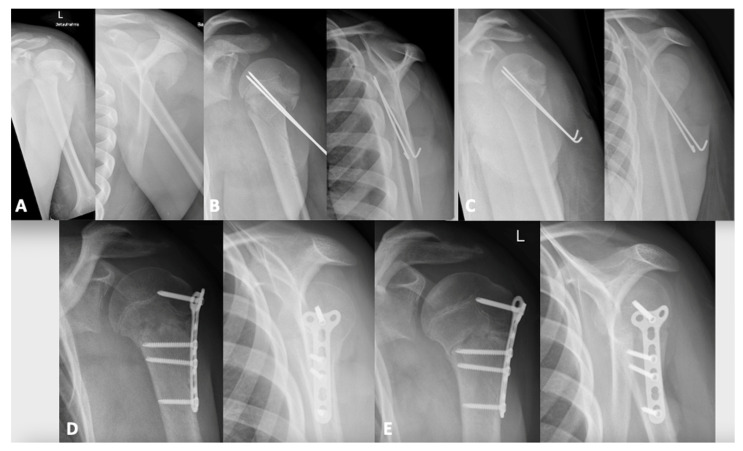
Chronologic radiographic images of Pat. N° 1. (**A**) Initial images after trauma with a posteriorly displaced proximal humeral fracture. (**B**) After closed reduction and transepiphyseal fixation. (**C**) X-ray 1 day after surgery shows posterior displacement in the Neer view (right side). (**D**) Open reduction and T-plate fixation. (**E**) X-ray follow-up 10 weeks after open reduction and plate-fixation with anatomical reduction and fracture healing.

The first patient (N° 1, Table 1, Figure 2) from this series was initially treated with a closed reduction and percutaneous pinning; due to secondary displacement, an open reduction and internal fixation was necessary.

**Figure 3:**
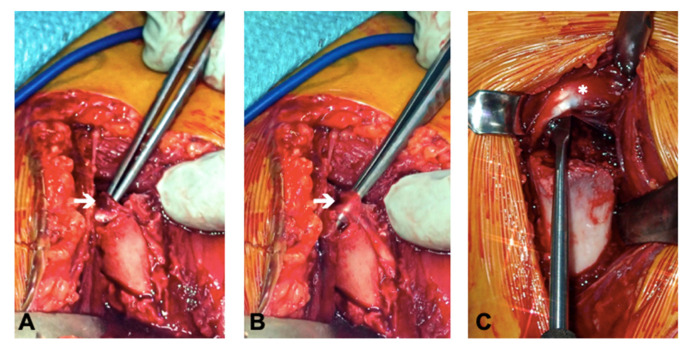
Intraoperative findings. (**A**) Entrapment of the long biceps tendon (white arrow) in the fracture gap (Pat. N° 1, see also Figure 2); (**B**) freed long biceps tendon (Pat. N° 1, see also Figure 2); (**C**) freed conjoint tendons (white asterisk) (Pat. N° 4, see also Figure 1).

For this patient, it was apparent that a closed reduction was impossible due to the interposition of soft tissue at the fracture site. Tissue entrapment was intraoperatively observed in all of the six cases (the long biceps tendon in two cases (Pat. N° 1 and 5), periosteal tissue in two cases (Pat. N° 3 and 6), a bony fragment in one case (Pat. N° 4), and both the long biceps tendon as well as the conjoint tendon in one case (Pat. N° 4)) (Figure 3).

The scheduled removal of the hardware was performed in all six patients. The implants were removed under general anesthesia as a day case procedure without difficulty at a mean time of 4.4 months after surgery (range 3–5.3 months).

The mean follow-up was 26 months (range: 22 months to 29 months) after fracture fixation. The Constant–Murley Shoulder and QuickDASH (including sport and music/work modules) scores are presented in (Table 1). The Constant–Murley score at the final follow-up was 97.5 (range 91 to 100) and the mean overall QuickDASH score (including sport and music/work) was 5.5 (range 0–20.8). Analyzing the subtypes of the QuickDASH score, we found a score of 3 for disability (range 0–10), 3.125 for sport and music (range 0–12.5) and 0 for work.

All fractures showed advanced radiological healing at the 10–12 weeks follow-up (Figure 1C,D). Postoperatively, there was no loss of reduction, residual deformity or screw migration.

**Figure 4:**
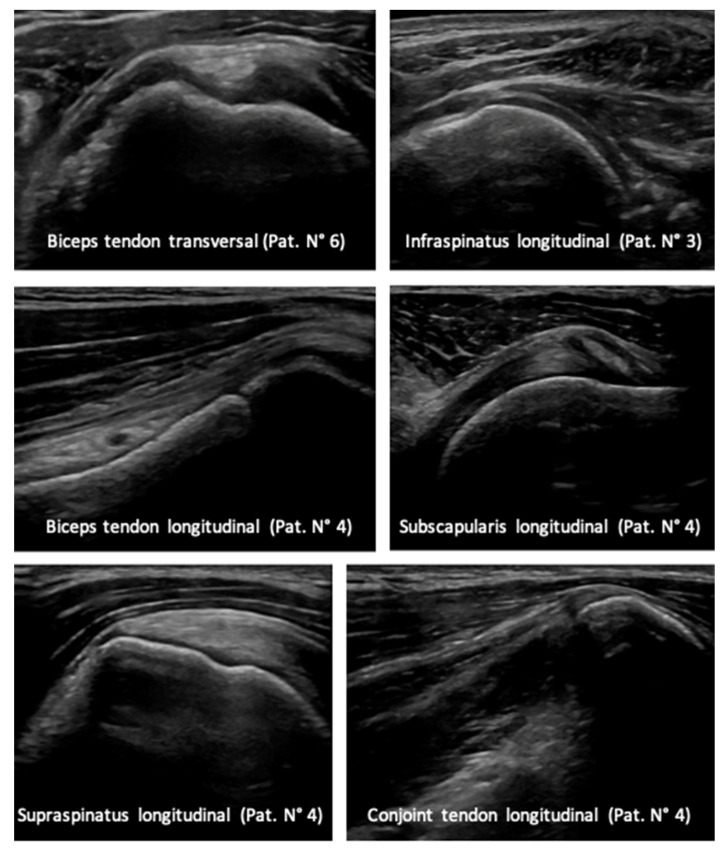
Sonographic scans 2 years postoperatively of the soft tissue surrounding the shoulder joint.

A sonographic examination of the shoulder two years postoperatively showed normal rotator cuff, long head of biceps and conjoint tendon (Figure 4).

No major complication was observed related to primary surgery or plate removal. None of the patients presented with vascular or neurological complications. All patients showed a rather apparent skin scar, known to appear frequently in this location [4]. Three out of six patients reported the scar to be esthetically disturbing. Two patients described a feeling of irritation at the operative scar, which they attributed to the plate irritation. This resolved completely after plate removal. At the 2-year follow-up, five patients were very satisfied and one was satisfied with the outcome.

## 4. Discussion

Recommended treatment for severely displaced proximal humeral fractures in children is closed reduction and percutaneous fixation by K-wires or intramedullary nailing [4,5,18]. We presented a series of six patients treated by an open reduction and plate fixation, in which either secondary displacement occurred, or closed reduction was unsatisfactory.

Two years postoperatively, we were able to demonstrate excellent shoulder function and preserved anatomical integrity of the entrapped tissues, namely, the biceps and conjoint tendon. To the best of our knowledge, this is the first report on the integrity of the interposed structures. It might well be questioned, whether these structures would have been intact, if a closed reduction had been accepted.

The first patient of this series (Figure 2) was initially treated with a closed reduction and percutaneous pinning. Due to secondary displacement, a revision with an open reduction and internal fixation was necessary caused by a biceps entrapment. Subsequently, we treated unreducible fractures by an open reduction and internal plate fixation and found tissue entrapment to be present in all cases.

**Figure 5:**
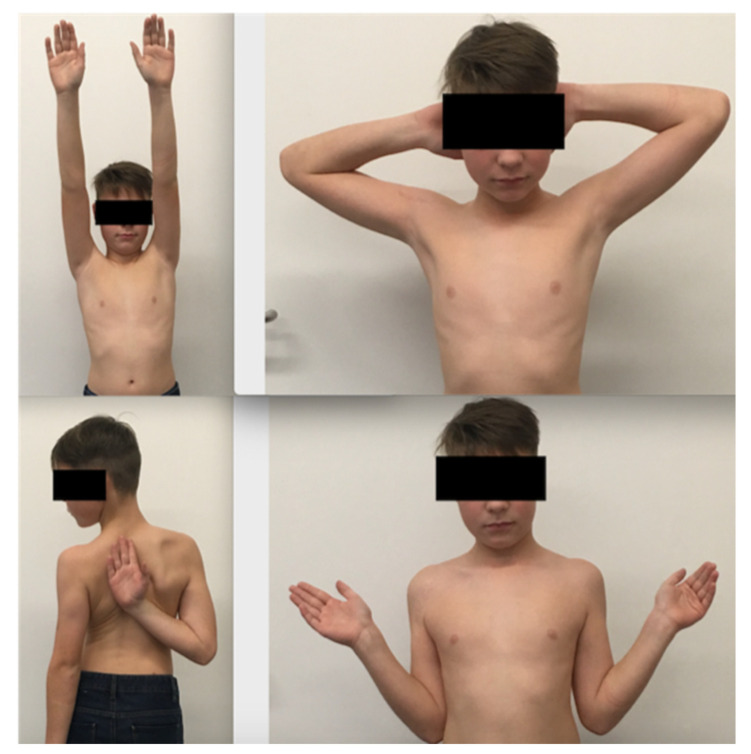
Range of motion after plate fixation of a proximal humerus fracture on the right site (Patient N° 6).

All fractures healed completely, and functional scores were excellent at a 2 year follow-up with symmetrical shoulder movement (Figure 5). The obstacle to reduction was, in most cases, the entrapped biceps tendon. In one case, the entrapped conjoint tendons inhibited reduction and in one other case periosteal tissue.

Dobbs et al. examined a subgroup of older adolescents and mentioned patients with irreducible fractures due to tissue entrapment which needed an open reduction [11].

The entrapment of the long head of the biceps tendon or periosteum was mentioned earlier but has not been identified as a major cause for irreducible fractures [4,5,11]. Lucas et al. did not find entrapment of the tendon of the long head of the biceps in the fracture site in four patients, which were assessed by magnetic resonance imaging.

In contrast, Bahrs et al. described open reduction in Neer III and IV fractures in 17 of their 31 patients. They found that in nine of these patients, the biceps tendon was entrapped in the fracture site. They concluded that a failed closed reduction should be interpreted as a possible soft tissue entrapment (most likely biceps tendon) and that these cases should be addressed with an open reduction and the removal of the entrapped structures [14].

Performing an open reduction, we could liberate the tissue interposed in the fracture side and achieve an anatomical reduction. To achieve a stable fixation of our reduction without crossing the physeal plate, we decided to use a T-plate fixation.

The use of a plate for an internal fixation after an open reduction has rarely been considered. In the aforementioned study of Bahrs et al., twelve patients were treated with a K-wire or screw fixation, and a plate fixation was used only in five patients. As early as 42 years ago, Weber et al. described treatment of severely displaced or irreducible infratubercular proximal humerus fractures by an open reduction and plate fixation without complications and symmetrical function of the shoulders.

In contrast to the generally preferred use of k-wires, a plate fixation avoids direct injury to the physis if screws are placed with the use of an image intensifier. If planned hardware removal is performed 3–6 months after initial surgery, tethering of the epiphyseal plate is not to be anticipated [14]. The drawback of the plate fixation, however, is the necessity of its removal at 3 months.

A plate fixation provides a stable fixation of the reduced fracture. Anatomical fracture healing is of upmost importance in adolescents (>12 years), where the remodeling capacity is limited [19]. In the present study, the average age of patients was 13 years. There is only limited data on the outcome of adolescent patients with this fractures [11].

In contrast to the plate fixation, any wire or elastic nail in metaphyseal and epiphyseal fractures will pass the epiphyseal plate and damage it to a certain degree. Excellent outcomes without limb shortening or axial deviation of the proximal humerus after K-wire or intramedullary nailing are reported [4,6,8]. Nevertheless, a physeal arrest and progressive deformity can be a potential risk of any crossing stabilization [20]. Peterson et al. reported on physeal injury in physeal fractures at three different sites (proximal humerus, distal humerus, distal femur) and recorded 100% premature closure in the three cases in which a K-wire internal fixation had passed the physis [21]. Intramedullary retrograde stabilization with ESIN has been recommended as the standard fixation method for proximal humeral fractures in children and adolescents, but this technique has some major drawbacks, such as nail penetration into the joint cavity, humeral head perforation, physeal damage due to multiple perforation and the displacement of the proximal fragment by pushing with the ESIN tips [4]. Zivanovic et al. observed complications in 5 of 16 patients: 2 humeral head perforations, 10° of residual varus deformation in 2 patients and difficulties in nail extraction in one patient. Similar complications were reported by other authors [8,22].

Our study has its limitations. First, the number of patients treated was small and general treatment recommendations could not be deducted. We did not have a control group that would demonstrate any superiority compared to other treatment strategies.

Second, due to the ethical restrictions, the study lacked a late radiographic follow-up at two years. However, as clinical results were excellent and ultrasound did not show any deformity of the tuberosities or the humeral head, it seems reasonable to state, that the growth plate was not subjected to any injury or tethering.

A drawback of our proposed treatment is the requirement of a second surgery.

We do agree with the generally accepted age and deformity-based decision making [4,5,18,23], but want to emphasize that tissue entrapment, which inhibits closed reduction, is very likely in Neer III and IV fractures, and may be underestimated in the literature so far.

## 5. Conclusions

If a proximal humeral fracture is not reducible by closed means, a tissue entrapment (most likely biceps tendon and conjoined tendon) has to be considered as an obstacle to the reduction. An open reduction and plate fixation not only yield excellent clinical results, but allow the functional and anatomical integrity of the entrapped tendons.

## Figures and Tables

**Table 1:** Outcome with 2-year follow-up after open reduction, internal plate fixation and early (mean 4 months postoperative) plate removal.

Case N°	Age	Sex	Reduction Method	Constant Score	QuickDASH Score				Subjective Outcome
					Total	Disability	Sport/Music	Work	
1	12 years	M	Closed/Open ^1^	98	0	0			Satisfied
2	14 years	F	Open	91	11.25	5	6.25	0	Very satisfied
3	14 years	M	Open	100	0	0	0		Very satisfied
4	14 years	M	Open	95	20.8	8.3	12.5	0	Very satisfied
5	16 years	M	Open	97	10	10			Very satisfied
6	8 years	M	Open	100	1	1	0		Very satisfied

^1^ secondary open reduction due to dislocation after closed reduction and percutaneous pinning (see also Figure 2).

## Data Availability

The data presented in this study are stored and openly available in the secretary bureau of the Department of Orthopaedics and Traumatology, Kantonsspital Baden, Im Ergel 1, 5404 Baden, Switzerland.
